# 7-Hy­droxy-1,2,3,4-tetra­hydro­quinolin-2-one dihydrate

**DOI:** 10.1107/S1600536812025263

**Published:** 2012-06-13

**Authors:** Qian-Shou Zong, Jian-Yi Wu

**Affiliations:** a College of Biology and Chemical Engineering, Jiaxing University, Jiaxing Zhejiang 314001, People’s Republic of China

## Abstract

The asymmetric unit of the title compound, C_9_H_9_NO_2_·2H_2_O, comprises two independent organic mol­ecules and four water mol­ecules of crystallization. The heterocyclic rings are not planar: in one mol­ecule, the C atom bearing the O atom and the adjacent methyl­ene C atom are displaced by 0.320 (3) and 0.677 (3) Å, respectively, from the other eight atoms of the fused ring system. Equivalent values of 0.243 (3) and 0.659 (3) Å apply to the second mol­ecule. In the crystal, the components are linked by N—H⋯O and O—H⋯O hydrogen bonds, forming a three-dimensional network.

## Related literature

For background to quinolin-2-ones as drugs, see: Braun *et al.* (2009*a* ▶,*b* ▶).
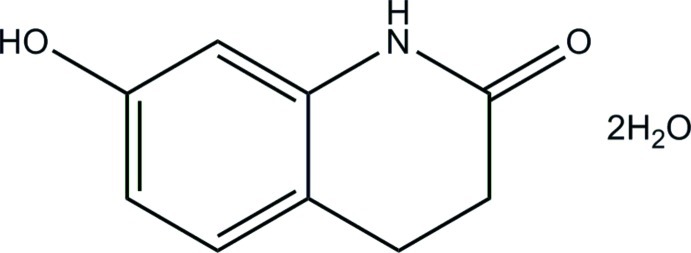


## Experimental

### 

#### Crystal data

C_9_H_9_NO_2_·2H_2_O
*M* *_r_* = 199.20
Orthorhombic, 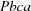

*a* = 15.4597 (16) Å
*b* = 12.7864 (12) Å
*c* = 20.312 (2) Å
*V* = 4015.1 (7) Å^3^
*Z* = 16
Mo *K*α radiation
μ = 0.10 mm^−1^
*T* = 298 K
0.20 × 0.17 × 0.15 mm

#### Data collection

Bruker APEXII CCD diffractometer
Absorption correction: multi-scan (*SADABS*; Sheldrick, 2004 ▶) *T*_min_ = 0.980, *T*_max_ = 0.985
36302 measured reflections
3659 independent reflections
3061 reflections with *I* > 2σ(*I*)
*R*_int_ = 0.058

#### Refinement

*R*[*F* ^2^ > 2σ(*F* ^2^)] = 0.084
*wR*(*F* ^2^) = 0.175
*S* = 1.30
3659 reflections
287 parameters
14 restraints
H atoms treated by a mixture of independent and constrained refinement
Δρ_max_ = 0.15 e Å^−3^
Δρ_min_ = −0.17 e Å^−3^

### 

Data collection: *APEX2* (Bruker, 2004 ▶); cell refinement: *SAINT* (Bruker, 2004 ▶); data reduction: *SAINT*; program(s) used to solve structure: *SHELXS97* (Sheldrick, 2008 ▶); program(s) used to refine structure: *SHELXL97* (Sheldrick, 2008 ▶); molecular graphics: *SHELXTL* (Sheldrick, 2008 ▶); software used to prepare material for publication: *SHELXTL*.

## Supplementary Material

Crystal structure: contains datablock(s) global, I. DOI: 10.1107/S1600536812025263/hb6830sup1.cif

Structure factors: contains datablock(s) I. DOI: 10.1107/S1600536812025263/hb6830Isup2.hkl

Supplementary material file. DOI: 10.1107/S1600536812025263/hb6830Isup3.cml

Additional supplementary materials:  crystallographic information; 3D view; checkCIF report

## Figures and Tables

**Table 1:** Hydrogen-bond geometry (Å, °)

*D*—H⋯*A*	*D*—H	H⋯*A*	*D*⋯*A*	*D*—H⋯*A*
O7—H7*B*⋯O5	0.85 (1)	1.95 (1)	2.800 (4)	174 (3)
O5—H5*A*⋯O2^i^	0.85 (1)	1.93 (1)	2.774 (3)	170 (3)
O8—H8*B*⋯O6^ii^	0.85 (1)	1.90 (1)	2.751 (4)	177 (4)
O7—H7*A*⋯O6^iii^	0.85 (1)	1.94 (1)	2.791 (4)	172 (3)
O5—H5*B*⋯O1^iv^	0.85 (1)	1.91 (1)	2.757 (3)	176 (3)
O8—H8*A*⋯O5^v^	0.85 (1)	1.95 (1)	2.790 (4)	169 (4)
N2—H2⋯O1^vi^	0.90 (1)	1.98 (1)	2.867 (3)	169 (3)
N1—H1⋯O3^vii^	0.90 (1)	1.99 (1)	2.895 (3)	177 (3)
O4—H4⋯O7^viii^	0.82	1.86	2.668 (4)	170
O2—H2*A*⋯O8^ix^	0.82	1.87	2.671 (4)	166
O6—H6*A*⋯O3	0.85 (1)	1.92 (1)	2.766 (3)	175 (4)

Symmetry codes: (i) 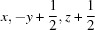; (ii) 
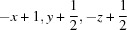
; (iii) 
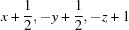
; (iv) 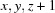; (v) 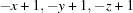; (vi) 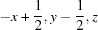; (vii) 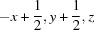; (viii) 
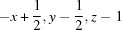
; (ix) 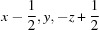.

**Fig. 1.**
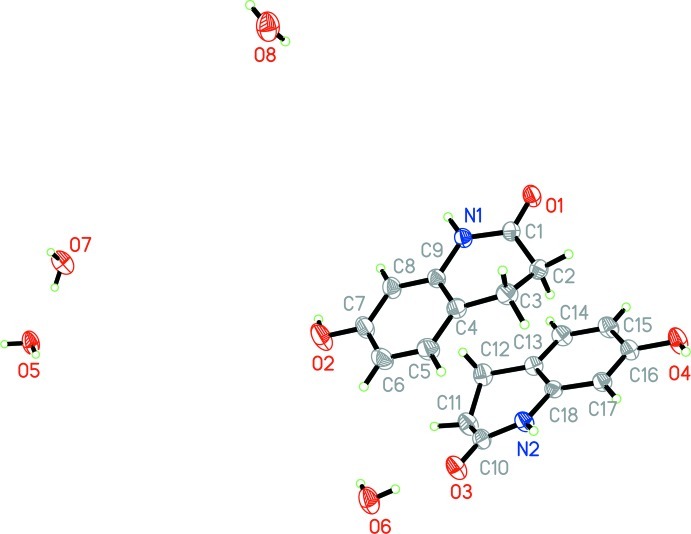
A view of the molecule of the title compound. Displacement ellipsoids are drawn at the 30% probability level.

**Fig. 2.**
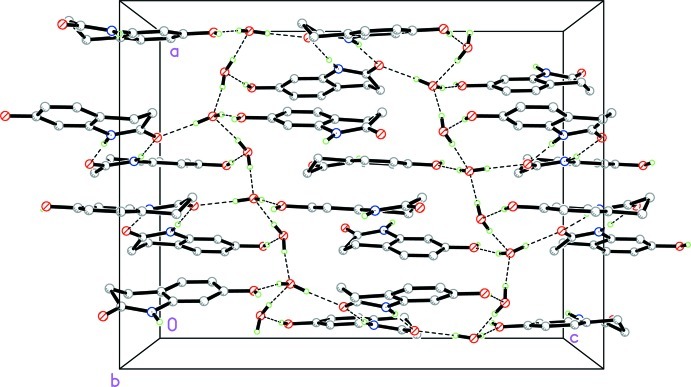
The crystal structure of the title compound, viewed along b axis. Hydrogen bonds are shown as dashed lines.

## supplementary crystallographic information

### Comment

7-Hydroxy-3,4-dihydro-1*H*-quinolin-2-one is an important intermediate for the preparation of non-typical antipsychotic drugs (Braun *et al.*, 2009*a*,*b*). In this paper, the author reports the structure of the compound.

The asymmetric unit of the title compound comprises two independent 7-hydroxy-3,4-dihydro-1*H*-quinolin-2-one moleclues and four water molecules of crystallization (Fig. 1). In the crystal, 7-hydroxy-3,4-dihydro-1*H*-quinolin-2-one moleclues are linked by water molecules through hydrogen bonds (Table 1), to form a 3D network (Fig. 2).

### Experimental

7-Hydroxy-3,4-dihydro-1*H*-quinolin-2-one was obtained from Jiaxing Taixin Pharmaceutical Chemical Co., Ltd, and recrystallized from aqueous solution as colourless blocks.

### Refinement

H1, H2 and the water H atoms were located from an electronic map and restrained with N—H, O—H, and H···H distances of 0.90 (1), 0.85 (1), and 1.37 (2) Å, respectively. All other H atoms were placed at calculated positions and refined using a riding model approximation, with C—H = 0.93 or 0.97 Å, O—H = 0.82 Å, and with *U*_iso_(H) = 1.2*U*_eq_(C) or 1.5*U*_eq_(O).

### Crystal data

**Table:**

C_9_H_9_NO_2_·2H_2_O	*D*_x_ = 1.318 Mg m^−^^3^
*M**_r_* = 199.20	Mo *K*α radiation, λ = 0.71070 Å
Orthorhombic, *P**b**c**a*	Cell parameters from 11198 reflections
*a* = 15.4597 (16) Å	θ = 2.8–25.3°
*b* = 12.7864 (12) Å	µ = 0.10 mm^−^^1^
*c* = 20.312 (2) Å	*T* = 298 K
*V* = 4015.1 (7) Å^3^	Block, colorless
*Z* = 16	0.20 × 0.17 × 0.15 mm
*F*(000) = 1696	

### Data collection

**Table:**

Bruker APEXII CCD diffractometer	3659 independent reflections
Radiation source: fine-focus sealed tube	3061 reflections with *I* > 2σ(*I*)
Graphite monochromator	*R*_int_ = 0.058
ω scan	θ_max_ = 25.4°, θ_min_ = 3.1°
Absorption correction: multi-scan (*SADABS*; Sheldrick, 2004)	*h* = −18→18
*T*_min_ = 0.980, *T*_max_ = 0.985	*k* = −15→15
36302 measured reflections	*l* = −24→22

### Refinement

**Table:**

Refinement on *F*^2^	Primary atom site location: structure-invariant direct methods
Least-squares matrix: full	Secondary atom site location: difference Fourier map
*R*[*F*^2^ > 2σ(*F*^2^)] = 0.084	Hydrogen site location: inferred from neighbouring sites
*wR*(*F*^2^) = 0.175	H atoms treated by a mixture of independent and constrained refinement
*S* = 1.30	*w* = 1/[σ^2^(*F*_o_^2^) + (0.0561*P*)^2^ + 1.7267*P*] where *P* = (*F*_o_^2^ + 2*F*_c_^2^)/3
3659 reflections	(Δ/σ)_max_ < 0.001
287 parameters	Δρ_max_ = 0.15 e Å^−^^3^
14 restraints	Δρ_min_ = −0.17 e Å^−^^3^

### Special details

**Table:**

Geometry. All esds (except the esd in the dihedral angle between two l.s. planes) are estimated using the full covariance matrix. The cell esds are taken into account individually in the estimation of esds in distances, angles and torsion angles; correlations between esds in cell parameters are only used when they are defined by crystal symmetry. An approximate (isotropic) treatment of cell esds is used for estimating esds involving l.s. planes.
Refinement. Refinement of F^2^ against ALL reflections. The weighted R-factor wR and goodness of fit S are based on F^2^, conventional R-factors R are based on F, with F set to zero for negative F^2^. The threshold expression of F^2^ > 2sigma(F^2^) is used only for calculating R-factors(gt) etc. and is not relevant to the choice of reflections for refinement. R-factors based on F^2^ are statistically about twice as large as those based on F, and R- factors based on ALL data will be even larger.

### Fractional atomic coordinates and isotropic or equivalent isotropic displacement parameters (Å^2^)

**Table:**

	*x*	*y*	*z*	*U*_iso_*/*U*_eq_	
H6B	0.044 (4)	0.0608 (19)	0.2751 (18)	0.17 (2)*	
H6A	0.049 (3)	0.012 (3)	0.2142 (6)	0.098 (14)*	
O1	0.36079 (14)	0.44183 (15)	−0.04017 (9)	0.0563 (6)	
O2	0.3059 (2)	0.18779 (19)	0.26380 (11)	0.0877 (9)	
H2A	0.3128	0.2485	0.2756	0.132*	
O3	0.06233 (14)	0.02384 (15)	0.12063 (10)	0.0586 (6)	
O4	0.06082 (19)	0.31032 (17)	−0.17569 (10)	0.0706 (7)	
H4	0.0693	0.2514	−0.1903	0.106*	
O5	0.29566 (19)	0.49405 (18)	0.83784 (11)	0.0750 (7)	
O6	0.0409 (2)	0.00260 (19)	0.25515 (12)	0.0779 (8)	
O7	0.4001 (2)	0.6303 (2)	0.76488 (14)	0.0886 (9)	
O8	0.8445 (2)	0.3721 (2)	0.18171 (13)	0.0800 (8)	
N1	0.35559 (16)	0.35016 (17)	0.05386 (11)	0.0456 (6)	
N2	0.07165 (16)	0.11997 (17)	0.02837 (11)	0.0465 (6)	
C1	0.33970 (18)	0.3603 (2)	−0.01053 (13)	0.0430 (7)	
C2	0.29447 (19)	0.2720 (2)	−0.04418 (14)	0.0488 (7)	
H2C	0.3106	0.2716	−0.0903	0.059*	
H2B	0.2325	0.2833	−0.0417	0.059*	
C3	0.3158 (2)	0.1665 (2)	−0.01435 (15)	0.0550 (8)	
H3A	0.2761	0.1144	−0.0314	0.066*	
H3B	0.3739	0.1463	−0.0271	0.066*	
C4	0.30961 (19)	0.1692 (2)	0.05908 (14)	0.0481 (7)	
C5	0.2861 (2)	0.0849 (2)	0.09787 (16)	0.0611 (9)	
H5	0.2708	0.0224	0.0776	0.073*	
C6	0.2847 (2)	0.0905 (3)	0.16567 (16)	0.0666 (9)	
H6	0.2689	0.0324	0.1905	0.080*	
C7	0.3070 (2)	0.1830 (2)	0.19654 (15)	0.0600 (9)	
C8	0.3304 (2)	0.2690 (2)	0.15928 (14)	0.0547 (8)	
H8	0.3453	0.3315	0.1797	0.066*	
C9	0.33155 (18)	0.2613 (2)	0.09142 (13)	0.0442 (7)	
C10	0.05833 (18)	0.1108 (2)	0.09337 (14)	0.0470 (7)	
C11	0.0364 (2)	0.2089 (2)	0.13002 (15)	0.0596 (8)	
H11A	0.0521	0.2004	0.1759	0.071*	
H11B	−0.0256	0.2204	0.1279	0.071*	
C12	0.0823 (2)	0.3036 (2)	0.10257 (15)	0.0559 (8)	
H12A	0.0577	0.3663	0.1218	0.067*	
H12B	0.1429	0.3007	0.1147	0.067*	
C13	0.07463 (18)	0.3091 (2)	0.02891 (14)	0.0453 (7)	
C14	0.0744 (2)	0.4010 (2)	−0.00691 (15)	0.0542 (8)	
H14	0.0774	0.4645	0.0154	0.065*	
C15	0.0699 (2)	0.4015 (2)	−0.07439 (15)	0.0569 (8)	
H15	0.0700	0.4647	−0.0971	0.068*	
C16	0.06517 (19)	0.3081 (2)	−0.10861 (14)	0.0500 (7)	
C17	0.06498 (18)	0.2143 (2)	−0.07431 (14)	0.0470 (7)	
H17	0.0615	0.1511	−0.0968	0.056*	
C18	0.06997 (18)	0.2159 (2)	−0.00617 (13)	0.0415 (6)	
H1	0.379 (2)	0.4051 (18)	0.0750 (15)	0.080*	
H2	0.085 (2)	0.0608 (16)	0.0065 (15)	0.080*	
H8A	0.7976 (12)	0.407 (2)	0.1786 (18)	0.080*	
H5B	0.317 (2)	0.476 (2)	0.8746 (9)	0.080*	
H7A	0.4461 (13)	0.595 (2)	0.7583 (18)	0.080*	
H8B	0.8793 (16)	0.412 (2)	0.2025 (16)	0.080*	
H5A	0.292 (2)	0.4385 (16)	0.8149 (13)	0.080*	
H7B	0.3651 (16)	0.591 (2)	0.7857 (17)	0.080*	

### Atomic displacement parameters (Å^2^)

**Table:**

	*U*^11^	*U*^22^	*U*^33^	*U*^12^	*U*^13^	*U*^23^
O1	0.0830 (15)	0.0451 (12)	0.0409 (11)	−0.0094 (11)	−0.0055 (10)	0.0034 (9)
O2	0.156 (3)	0.0627 (15)	0.0439 (14)	−0.0189 (17)	−0.0015 (15)	0.0082 (11)
O3	0.0874 (16)	0.0441 (12)	0.0443 (12)	0.0079 (11)	0.0094 (10)	0.0045 (9)
O4	0.115 (2)	0.0526 (13)	0.0444 (13)	0.0070 (14)	−0.0001 (12)	0.0085 (10)
O5	0.119 (2)	0.0561 (14)	0.0498 (14)	0.0043 (14)	−0.0149 (14)	−0.0023 (11)
O6	0.130 (2)	0.0563 (14)	0.0474 (14)	0.0020 (15)	−0.0051 (15)	−0.0005 (12)
O7	0.124 (3)	0.0710 (18)	0.0709 (18)	0.0040 (16)	0.0160 (17)	0.0059 (14)
O8	0.108 (2)	0.0669 (16)	0.0649 (16)	0.0013 (15)	−0.0148 (15)	−0.0050 (13)
N1	0.0596 (16)	0.0379 (13)	0.0392 (13)	−0.0049 (11)	−0.0025 (11)	0.0006 (10)
N2	0.0634 (16)	0.0349 (13)	0.0413 (14)	0.0017 (11)	0.0053 (11)	−0.0006 (10)
C1	0.0461 (16)	0.0432 (16)	0.0395 (16)	0.0036 (13)	0.0015 (12)	−0.0038 (12)
C2	0.0556 (17)	0.0494 (17)	0.0413 (16)	−0.0053 (14)	−0.0023 (13)	−0.0060 (13)
C3	0.071 (2)	0.0421 (16)	0.0520 (19)	−0.0070 (15)	0.0026 (15)	−0.0079 (13)
C4	0.0549 (18)	0.0424 (16)	0.0470 (17)	−0.0011 (13)	−0.0012 (14)	−0.0023 (13)
C5	0.082 (2)	0.0413 (17)	0.060 (2)	−0.0107 (16)	−0.0020 (17)	−0.0019 (14)
C6	0.092 (3)	0.0486 (19)	0.059 (2)	−0.0148 (17)	−0.0024 (18)	0.0111 (15)
C7	0.085 (2)	0.0513 (19)	0.0436 (18)	−0.0088 (17)	−0.0035 (16)	0.0070 (14)
C8	0.074 (2)	0.0465 (17)	0.0437 (17)	−0.0039 (15)	−0.0055 (15)	−0.0001 (13)
C9	0.0499 (16)	0.0404 (15)	0.0423 (16)	−0.0009 (13)	−0.0009 (13)	0.0018 (12)
C10	0.0536 (18)	0.0463 (17)	0.0412 (16)	0.0042 (14)	0.0030 (13)	0.0000 (13)
C11	0.082 (2)	0.0498 (17)	0.0472 (18)	0.0075 (16)	0.0098 (16)	−0.0024 (14)
C12	0.074 (2)	0.0450 (17)	0.0490 (18)	0.0065 (15)	−0.0014 (16)	−0.0089 (13)
C13	0.0483 (16)	0.0404 (15)	0.0471 (16)	0.0055 (13)	0.0016 (13)	−0.0021 (12)
C14	0.069 (2)	0.0369 (16)	0.0571 (19)	0.0032 (14)	0.0042 (15)	−0.0038 (13)
C15	0.073 (2)	0.0368 (16)	0.060 (2)	0.0052 (15)	0.0011 (16)	0.0072 (14)
C16	0.0597 (19)	0.0469 (16)	0.0435 (16)	0.0026 (14)	0.0017 (14)	0.0049 (13)
C17	0.0560 (18)	0.0382 (15)	0.0469 (17)	−0.0011 (13)	0.0015 (13)	−0.0012 (12)
C18	0.0449 (16)	0.0377 (15)	0.0419 (16)	−0.0012 (12)	0.0018 (12)	0.0018 (12)

### Geometric parameters (Å, º)

**Table:**

O1—C1	1.247 (3)	C3—H3B	0.9700
O2—C7	1.368 (4)	C4—C5	1.384 (4)
O2—H2A	0.8200	C4—C9	1.390 (4)
O3—C10	1.244 (3)	C5—C6	1.379 (4)
O4—C16	1.364 (3)	C5—H5	0.9300
O4—H4	0.8200	C6—C7	1.382 (4)
O5—H5B	0.851 (10)	C6—H6	0.9300
O5—H5A	0.851 (10)	C7—C8	1.383 (4)
O6—H6B	0.850 (10)	C8—C9	1.382 (4)
O6—H6A	0.850 (10)	C8—H8	0.9300
O7—H7A	0.854 (10)	C10—C11	1.498 (4)
O7—H7B	0.853 (10)	C11—C12	1.509 (4)
O8—H8A	0.853 (10)	C11—H11A	0.9700
O8—H8B	0.854 (10)	C11—H11B	0.9700
N1—C1	1.337 (3)	C12—C13	1.502 (4)
N1—C9	1.419 (3)	C12—H12A	0.9700
N1—H1	0.902 (10)	C12—H12B	0.9700
N2—C10	1.341 (3)	C13—C14	1.382 (4)
N2—C18	1.414 (3)	C13—C18	1.390 (4)
N2—H2	0.902 (10)	C14—C15	1.372 (4)
C1—C2	1.494 (4)	C14—H14	0.9300
C2—C3	1.516 (4)	C15—C16	1.384 (4)
C2—H2C	0.9700	C15—H15	0.9300
C2—H2B	0.9700	C16—C17	1.387 (4)
C3—C4	1.495 (4)	C17—C18	1.386 (4)
C3—H3A	0.9700	C17—H17	0.9300
			
C7—O2—H2A	109.5	C9—C8—C7	119.5 (3)
C16—O4—H4	109.5	C9—C8—H8	120.3
H5B—O5—H5A	106 (2)	C7—C8—H8	120.3
H6B—O6—H6A	109 (2)	C8—C9—C4	121.9 (3)
H7A—O7—H7B	107 (2)	C8—C9—N1	118.8 (2)
H8A—O8—H8B	105 (2)	C4—C9—N1	119.2 (2)
C1—N1—C9	123.8 (2)	O3—C10—N2	120.6 (2)
C1—N1—H1	118 (2)	O3—C10—C11	122.6 (3)
C9—N1—H1	118 (2)	N2—C10—C11	116.8 (2)
C10—N2—C18	124.2 (2)	C10—C11—C12	112.4 (2)
C10—N2—H2	117 (2)	C10—C11—H11A	109.1
C18—N2—H2	119 (2)	C12—C11—H11A	109.1
O1—C1—N1	120.4 (2)	C10—C11—H11B	109.1
O1—C1—C2	122.2 (2)	C12—C11—H11B	109.1
N1—C1—C2	117.4 (2)	H11A—C11—H11B	107.8
C1—C2—C3	112.9 (2)	C13—C12—C11	111.6 (3)
C1—C2—H2C	109.0	C13—C12—H12A	109.3
C3—C2—H2C	109.0	C11—C12—H12A	109.3
C1—C2—H2B	109.0	C13—C12—H12B	109.3
C3—C2—H2B	109.0	C11—C12—H12B	109.3
H2C—C2—H2B	107.8	H12A—C12—H12B	108.0
C4—C3—C2	111.4 (2)	C14—C13—C18	117.3 (3)
C4—C3—H3A	109.4	C14—C13—C12	124.4 (3)
C2—C3—H3A	109.4	C18—C13—C12	118.3 (2)
C4—C3—H3B	109.4	C15—C14—C13	122.0 (3)
C2—C3—H3B	109.4	C15—C14—H14	119.0
H3A—C3—H3B	108.0	C13—C14—H14	119.0
C5—C4—C9	117.0 (3)	C14—C15—C16	120.0 (3)
C5—C4—C3	124.5 (3)	C14—C15—H15	120.0
C9—C4—C3	118.4 (3)	C16—C15—H15	120.0
C6—C5—C4	122.2 (3)	O4—C16—C15	119.1 (3)
C6—C5—H5	118.9	O4—C16—C17	121.3 (3)
C4—C5—H5	118.9	C15—C16—C17	119.6 (3)
C5—C6—C7	119.6 (3)	C18—C17—C16	119.2 (3)
C5—C6—H6	120.2	C18—C17—H17	120.4
C7—C6—H6	120.2	C16—C17—H17	120.4
O2—C7—C6	119.2 (3)	C17—C18—C13	121.9 (2)
O2—C7—C8	120.9 (3)	C17—C18—N2	118.9 (2)
C6—C7—C8	119.8 (3)	C13—C18—N2	119.2 (2)

### Hydrogen-bond geometry (Å, º)

**Table:**

*D*—H···*A*	*D*—H	H···*A*	*D*···*A*	*D*—H···*A*
O7—H7*B*···O5	0.85 (1)	1.95 (1)	2.800 (4)	174 (3)
O5—H5*A*···O2^i^	0.85 (1)	1.93 (1)	2.774 (3)	170 (3)
O8—H8*B*···O6^ii^	0.85 (1)	1.90 (1)	2.751 (4)	177 (4)
O7—H7*A*···O6^iii^	0.85 (1)	1.94 (1)	2.791 (4)	172 (3)
O5—H5*B*···O1^iv^	0.85 (1)	1.91 (1)	2.757 (3)	176 (3)
O8—H8*A*···O5^v^	0.85 (1)	1.95 (1)	2.790 (4)	169 (4)
N2—H2···O1^vi^	0.90 (1)	1.98 (1)	2.867 (3)	169 (3)
N1—H1···O3^vii^	0.90 (1)	1.99 (1)	2.895 (3)	177 (3)
O4—H4···O7^viii^	0.82	1.86	2.668 (4)	170
O2—H2*A*···O8^ix^	0.82	1.87	2.671 (4)	166
O6—H6*A*···O3	0.85 (1)	1.92 (1)	2.766 (3)	175 (4)

Symmetry codes: (i) *x*, −*y*+1/2, *z*+1/2; (ii) −*x*+1, *y*+1/2, −*z*+1/2; (iii) *x*+1/2, −*y*+1/2, −*z*+1; (iv) *x*, *y*, *z*+1; (v) −*x*+1, −*y*+1, −*z*+1; (vi) −*x*+1/2, *y*−1/2, *z*; (vii) −*x*+1/2, *y*+1/2, *z*; (viii) −*x*+1/2, *y*−1/2, *z*−1; (ix) *x*−1/2, *y*, −*z*+1/2.
